# Gene expression profiling analysis of osteosarcoma cell lines

**DOI:** 10.3892/mmr.2015.3958

**Published:** 2015-06-18

**Authors:** LU SUN, JIE LI, BING YAN

**Affiliations:** Department of Orthopedics, Shandong Chinese Medical Hospital, Jinan, Shandong 250014, P.R. China

**Keywords:** osteosarcoma, protein-protein interaction network, differentially expressed genes, module analysis

## Abstract

Osteosarcoma (OS) is the most common type of primary bone malignancy and has a poor prognosis. To investigate the mechanisms of osteosarcoma, the present analyzed the GSE28424 microarray. GSE28424 was downloaded from the Gene Expression Omnibus, and included a collective of 19 OS cell lines and four normal bone cell lines, which were used as controls. Subsequently, the differentially expressed genes (DEGs) were screened using the Limma package in Bioconductor. Gene Ontology (GO) and pathway enrichment analysis of the DEGs was performed using the Database for Annotation, Visualization and Integrated Discovery, interactions between the proteins encoded by the DEGs were identified using STRING, and the protein-protein interaction (PPI) network was visualized using Cytoscape. In addition, modular analysis of the PPI network was performed using the Clique Percolation Method (CPM) in CFinder. A total of 1,170 DEGs were screened, including 530 upreguated and 640 downregulated genes. The enriched functions included organelle fission, immune response and response to wounding. In addition, RPL8 was observed to be involved with the ribosomal pathway in module A of the PPI network of the DEGs. PLCG1, SYK and PLCG2 were also involved in the B-cell receptor signaling pathway in module B and the Fc-epsilon RI signaling pathway in module C. In addition, AURKA (degree=39), MAD2L1 (degree=38), CDCA8 (degree=38), BUB1 (degree=37) and MELK (degree=37) exhibited higher degrees of connectivity in module F. The results of the present study suggested that the RPL8, PLCG1, PLCG2, SYK, MAD2L1, AURKA, CDCA8, BUB1 and MELK genes may be involved in OS.

## Introduction

Osteosarcoma (OS) is the most common type of primary bone malignancy derived from primitive mesenchymal cells (1). OS originates predominantly from bone and rarely from soft tissue (2). With a high degree of malignancy, rapid progression and poor prognosis, OS occurs predominantly in teenagers, and, in adolescents between the ages of 15 and 19 years, OS comprises 15% of all types of solid extra cranial cancer (3,4).

Several studies have been performed to investigate the molecular mechanisms underlying OS. For example, increased expression levels of vascular endothelial growth factor A (VEGFA) have been reported to induce metastasis and OS development (5–7), the major angiogenic factors, VEGF165 and VEGF189, may be critical for neovascularisation in OS (8), and pigment epithelium-derived factor (PEDF) not only induces apoptotic cell death in OS cells, but also suppress the expression of VEGF, resulting in the inhibition of tumor angiogenesis (9). Alterations in p53 have also been found in uncultured OS samples, with a frequency ranging between 18 and 42%, indicating that p53 may be involved in the pathogenesis of OS (10,11). In the context of bone morphogenetic protein (BMP)/small mothers of decapentaplegic (SMAD) signaling, runt-related transcription factor 2 (Runx2) can act as an inducer of apoptosis and suppressor of growth in OS and normal osteoblasts (12). There exists an important role for human epidermal growth factor receptor 2 (HER2) in the promotion of metastatic potential in OS and in aggressive tumor growth (13).

In 2012, Namløs *et al* (14) used an integrative microarray approach to analyze genome-wide mRNA expression patterns between a panel of 19 EuroBoNeT OS cell lines and four normal bone cell lines, according to the cut-off point of false discovery rate (FDR) <0.05, and obtained 8,982 mRNA probes, which were significantly differently expressed between the two groups. Using the same data used by Namløs *et al* (14), the present study aimed to further screen for differentially expressed genes (DEGs) using a more strict threshold of FDR<0.01 and |log2fold-change (FC)|>1, and to analyze the potential functions of the DEGs using Gene Ontology (GO) functional analysis and Kyoto Encyclopedia of Genes and Genomes (KEGG) pathway enrichment analysis. In addition, the present study aimed to construct a protein-protein interaction (PPI) network of these DEGs and screen the modules of the PPI network to identify the interactions/association between the DEGs.

## Materials and methods

### Microarray data

The expression profile of GSE28424, deposited by Namløs *et al* (14) was downloaded from the Gene Expression Omnibus (GEO; http://www.ncbi.nlm.nih.gov/geo/), which was based on the platform of the GPL13376 Illumina HumanWG-6 v2.0 expression beadchip (Illumina, San Diego, CA, USA). The GSE28424 dataset included a collective of 19 OS cell lines and four normal bone cell lines, of which the latter were used as controls.

### DEGs screening

Following downloading of the GSE28424 data, the microarray data was normalized to a linear model of box plots of log2[(perfect match probe value)-MM (mismatch probe value)] using quantile normalization (15). Subsequently, the DEGs between the OS patients and normal control cell lines were analyzed using the linear models for microarray data (Limma) package in Bioconductor (http://www.bioconductor.org/packages/release/bioc/html/limma.html) (16). The adjusted P-value (FDR)<0.01 and |log2FC|>1 were used as the cut-off criteria.

### Functional analysis and pathway enrichment analysis

As an integrated and high-throughput data-mining environment, the Database for Annotation, Visualization and Integrated Discovery (DAVID; http://david.abcc.ncifcrf.gov/) analyzes gene lists from high-throughput genomic experiments (17). The aim of Gene Ontology (GO; http://www.geneontology.org/) is to provide access to extensive documentation and perform functional analyses (18) KEGG (http://www.genome.ad.jp/kegg/) is a comprehensive database resource, which consists of chemical information, genomic information and systems information (19). Using the DAVID online tool, GO functional analysis and KEGG pathway enrichment analysis were performed for the DEGs. In addition, the GO functional enrichment analyses was focussed predominantly on biological process (BP). P<0.05 was used as the cut-off criterion.

### PPI network and module construction

The Search Tool for the Retrieval of Interacting Genes (STRING) online software (http://string-db.org/) (20) was used to identify interactions/associations between the proteins encoded by the DEGs, and a combined score >0.7 was used as the cut-off criterion. Subsequently, Cytoscape software (http://cytoscape.org/download_old_versions.html) (21) was used to visualize the PPI network and the Clique Percolation Method (CPM) was used in CFinder software (http://cfinder.org/) (22) to screen the modules of the PPI network. The clique value (k) was set to six.

## Results

### DEG analysis

A total of 1,170 DEGs were screened, which included 530 upregulated genes and 640 downregulated genes. There were a higher number of downregulated genes, compared with upregulated genes.

### Functional analysis and pathway enrichment analysis

The top 10 most enriched GO functions for the DEGs are listed in Table I. For the upregulated genes, the enriched functions in the BP category included organelle fission (P=1.19E-09), cell cycle process (P=3.90E-09) and nuclear division (P=8.03E-09). For the downregulated genes, the enriched functions in the BP category included immune response (P=5.15E-32), defense response (P=2.69E-28) and response to wounding (P=1.40E-15).

The top 10 enriched KEGG pathways for the DEGs are listed in Table II. For the upregulated genes, the enriched pathways included steroid biosynthesis (P=0.0023), ribosome (P=0.0294) and aminoacyl-tRNA biosynthesis (P=0.0125). For the downregulated genes, the enriched pathways included cell adhesion molecules (CAMs, P=3.74E-05), hematopoietic cell lineage (P=1.05E-04) and natural killer cell-mediated cytotoxicity (P=1.36E-04).

### PPI network and module construction

**Figure 1:**
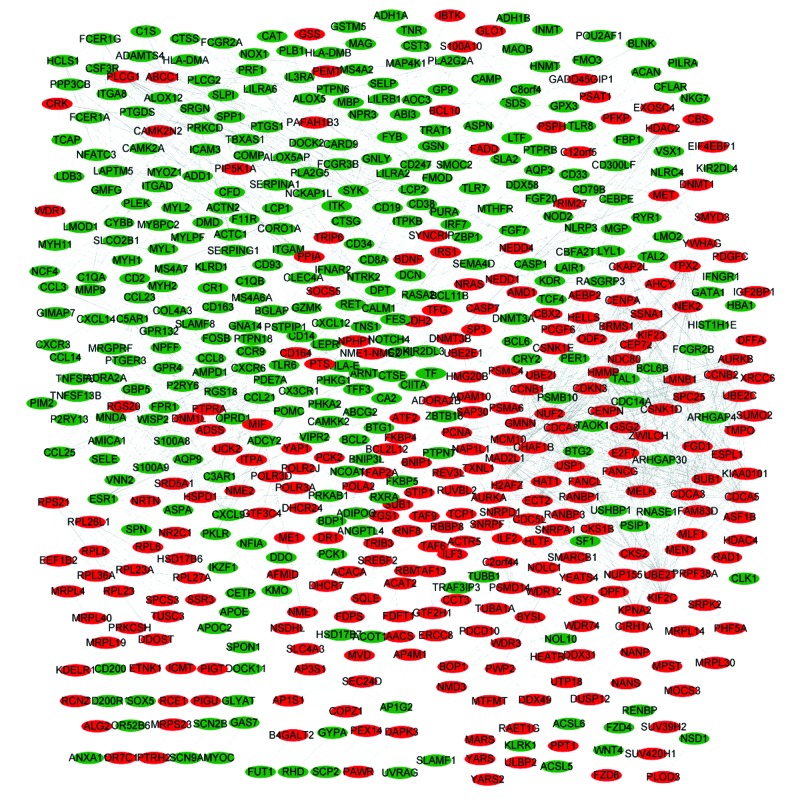
Protein-protein interaction network of the differentially expressed genes. The red and green circles represent for the upregulated and downregulated genes, respectively.

**Figure 2:**
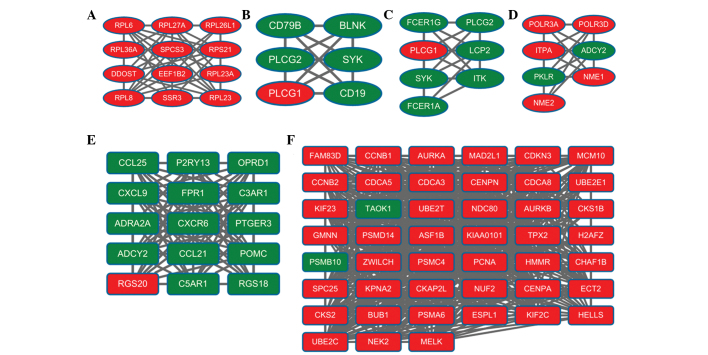
The top six modules (A-F) obtained from the protein-protein interaction network of the differentially expressed genes. The circular and square nodes represent proteins and the grey lines represent interactions. Red circles indicate upregulated genes and green circles represent downregulated genes.

The PPI network of the DEGs was revealed to have 590 nodes and 1,888 interactions (Fig. 1). A total of 10 modules were obtained from the PPI network, of which the top six modules (modules A-F) are shown in Fig. 2. The enriched pathways for the DEGs in the six modules are listed in Table III. Module A had 12 nodes and 50 interactions. In this module, all the DEGs were upregulated genes. The enriched KEGG pathways for DEGs in module A included ribosome (P=2.65E-12), which involved ribosomal protein L8 (RPL8).

Module B had six nodes and 15 interactions. In this module, the number of upregulated genes was considerably lower than the number of downregulated genes. For the DEGs in module B, the enriched KEGG pathways included the B-cell receptor signaling pathway (P=2.16E-07), Fc epsilon RI signaling pathway (P=0.0023) and natural killer cell-mediated cytotoxicity (P=0.0064). In module B, the B -cell receptor signaling pathway involved phospholipase Cγ1 (PLCγ1), spleen tyrosine kinase (SYK) and phospholipase Cγ2 (PLCγ2).

Module C had seven nodes and 20 interactions. In this module, the number of upregulated genes was markedly lower than the number of downregulated genes. The enriched KEGG pathways for the DEGs in module C included the Fc epsilon RI signaling pathway (P=4.43E-09), FcγR-mediated phagocytosis (P=0.0049) and leukocyte transendothelial migration (P=0.0075). In module C, the Fc-epsilon RI signaling pathway also involved PLCγ1, SYK and PLCγ2.

Module D had seven nodes and 20 interactions. In this module, the number of upregulated genes was higher than the number of downregulated genes. For the DEGs in module D, the enriched KEGG pathways included purine metabolism (P=2.31E-08), pyrimidine metabolism (P=6.15E-05) and the cytosolic DNA-sensing pathway (P=0.0529). The purine metabolism pathway involved adenylate cyclase 2 (ADCY2), NME/NM23 nucleoside diphosphate kinase 1 (NME1) and pyruvate kinase, liver and red blood cell (PKLR).

Module E had 15 nodes and 102 interactions. In this module, the number of upregulated genes was significantly lower than the number of downregulated genes. The enriched KEGG pathways for DEGs in module E included neuroactive ligand-receptor interaction (P=1.10E-05), chemokine signaling pathway (P=6.95E-04) and cytokine-cytokine receptor interaction (P=0.0210). The neuroactive ligand-receptor interaction pathway involved complement component 5a receptor 1 (C5AR1), prostaglandin E receptor 3 (PTGER3) and formyl peptide receptor 1 (FPR1).

Module F had 45 nodes and 609 interactions. In this module, the number of upregulated genes was significantly more than the number of downregulated genes. For the DEGs in module F, the enriched KEGG pathways included oocyte meiosis (P=1.56E-05), cell cycle (P=2.91E-05) and progesterone-mediated oocyte maturation (P=0.0022). Notably, cyclin B1 (CCNB1; degree=40), aurora kinase A (AURKA; degree=39), MAD2 mitotic arrest defective-like 1 (MAD2L1; degree=38), cell division cycle associated 8 (CDCA8; degree=38), budding uninhibited by benzimidazoles 1 (BUB1; degree=37) and maternal embryonic leucine zipper kinase (MELK; degree=37) exhibited higher degrees of connectivity. In addition, several interactive associations were identified, including MAD2L1-AURKA, MAD2L1-CDCA8, MAD2L1-BUB1 and MAD2L1-MELK.

## Discussion

In the present study, a total of 1,170 DEGs were screened, including 530 upregulated genes and 640 downregulated genes. The enriched pathways for the DEGs included steroid biosynthesis and ribosome. In particular, AURKA (degree=39), MAD2L1 (degree=38), CDCA8 (degree=38), BUB1 (degree=37) and MELK (degree=37) exhibited higher degrees of connectivity in module F of the PPI network of the DEGs.

As a member of the L2P family of ribosomal proteins, RPL8 is a component of the 60S ribosomal subunit in eucaryotic cells (23). Amplification of RPL8 may be associated with the pathogenesis of OS (24). RPL8 can respond to the chemotherapy in conventional OS, and it may be beneficial in the assessment at diagnosis and for stratifying participants of randomized trials (25). A daily-repeated ribosome biogenesis inhibition can result in progressive reduction of ribosome content and extinction of protein- and p53-deficient human OS cell lines (26). In module A, the enriched ribosome pathway involved RPL8, indicating that RPL8 may be involved in OS.

PLC is important in mediating intracellular signal transduction (27). As a member of the PLC family, PLCγ1 is upregulated in several cancer cell lines and cancer tissues (28). It is also reported that PLCγ1 promotes the invasion of several types of tumor (29-31). PLCγ2 translocation is essential in transmitting TPA signal to PKCα and PKCα can directly promote the apoptosis of human cancer cell lines, thus, PLCγ2 is critical in the process of inducting apoptosis (32). The SYK gene can be reactivated by inhibition of DNA promoter methylation in human cancer and may act as a tumor suppressor (33). The B-cell receptor signaling pathway has been correlated with OS (34). In module B in the present study, the B-cell receptor signaling pathway involved PLCγ1, SYK and PLCγ2. The Fc-epsilon RI signaling pathway involves multiple component genes that are altered at the chromosome level, and may be associated with the pathogenesis of OS (5). In module C, the Fc-epsilon RI signaling pathway also involved PLCγ1, SYK and PLCγ2. Thus, it was hypothesized that PLCγ1, SYK and PLCγ2 may be closely correlated with OS. In addition, in modules B and C, the upregulated PLCγ1 was observed to have an interactive association with down-regulated SYK and PLCγ2, indicating that PLCγ1 may also be involved in OS by mediating SYK and PLCγ2.

The overexpression of MAD2 can be caused by retinoblastoma pathway inactivation and is associated with carcinogenesis (35). The expression of MAD2 is upregulated in human OS, and its overexpression is involved in earlier metastasis and poorer survival rates in patients with human OS (36). Knockdown of MAD2 can induce OS cell apoptosis, involving the cleavage of Rad21 (37). This suggests that MAD2L1 may be closely correlated with OS. The mitotic checkpoint gene, BUB1, may also drive tumor metastasis and progression (38). CDCA8 and aurora kinase B (AURKB) are overexpressed in tumor cells (39,40) and selective suppression of the CDCA8-AURKB pathway may be a promising therapeutic strategy in the treatment of cancer (41). MELK is a protein kinase and candidate oncoprotein, which is dysregulated in several types of cancer (42–44), as well as being involved in resistance to apoptosis (43). In module F in the present study, MAD2L1 had interactive associations seperately with AURKA, CDCA8, BUB1 and MELK, suggesting that MAD2L1 may also be involved in OS by mediating AURKA, CDCA8, BUB1 and MELK.

In conclusion, the present study performed a comprehensive bioinformatics analysis of genes, which may be involved in OS. A total of 1,170 DEGs were screened, which including 530 upregulated genes and 640 downregulated genes. The results of the subsequent analyses suggested that RPL8, PLCγ1, PLCγ2, SYK, MAD2L1, AURKA, CDCA8, BUB1 and MELK may be correleted with OS. However, further investigations are required to elucidate their mechanisms of action in OS.

## Figures and Tables

**Table I:** The top 10 most enriched GO functions for the differentially expressed genes.

Gene	Category	ID	Term	Number of genes	Examples of genes	P-value
Upregulated	BP	GO:0048285	Organelle fission	56	KIF23, CEP72	1.19E-09
		GO:0022402	Cell cycle process	45	BCAT1, KIF23	3.90E-09
		GO:0000280	Nuclear division	26	KIF23, CEP72	8.03E-09
		GO:0000278	Mitotic cell cycle	35	KIF23, BCAT1	4.44E-09
		GO:0007067	Mitosis	26	MAD2L1, CCNB2	8.03E-09
Downregulated	BP	GO:0006955	Immune response	100	AQP9, TLR2	5.15E-32
		GO:0006952	Defense response	89	KIR2DL3, CD300LB	2.69E-28
		GO:0006954	Inflammatory response	53	MBP, CD96	4.47E-19
		GO:0002684	Positive regulation of immune system process	43	CCL23, HRH2	3.47E-17
		GO:0009611	Response to wounding	63	FGF7, ACVRL1	1.40E-15

GO, Gene Ontology; BP, biological process.

**Table II:** The top 10 most enriched Kyoto Encyclopedia of Genes and Genomes pathways for the differentially expressed genes.

Gene	Name	Number of genes	Examples of genes	P-value
Upregulated	hsa00100:Steroid biosynthesis	5	SQLE, DHCR7	0.0023
	hsa00270:Cysteine and methionine metabolism	6	AHCY, DNMT1	0.0056
	hsa03040:Spliceosome	11	SNRPA1, MAGOH	0.0110
	hsa00970:Aminoacyl-tRNA biosynthesis	6	YARS, NARS2	0.0125
	hsa03010:Ribosome	8	RPL6, RPL8	0.0294
Downregulated	hsa04514:Cell adhesion molecules	20	HLA-DQB1, F11R	3.74E-05
	hsa05310:Asthma	9	CD2, SELE	7.43E-05
	hsa04640:Hematopoietic cell lineage	15	IL1R2, CR1	1.05E-04
	hsa05332:Graft-versus-host disease	10	PRF1, KIR2DL3	1.21E-04
	hsa04650:Natural killer cell mediated cytotoxicity	19	PRF1, PTPN6	1.36E-04

**Table III:** Enriched Kyoto Encyclopedia of Genes and Genomes pathways for differentially expressed genes in modules A-F.

Module	Name	Number of genes	Examples of genes	P-value
A	hsa03010:Ribosome	8	RPL6, RPL8	2.65E-12
B	hsa04662:B cell receptor signaling pathway	5	PLCγ2, SYK	2.16E-07
	hsa04664:Fc epsilon RI signaling pathway	3	PLCγ1, PLCγ2, SYK	0.0023
	hsa04666:Fc gamma R-mediated phagocytosis	3	PLCγ1, PLCγ2, SYK	0.0033
	hsa04650:Natural killer cell mediated cytotoxicity	3	PLCγ1, PLCγ2, SYK	0.0064
	hsa05340:Primary immunodeficiency	2	CD19, BLNK	0.0340
	hsa04664:Fc epsilon RI signaling pathway	6	PLCγ1, PLCγ2, SYK	4.43E-09
	hsa04650:Natural killer cell mediated cytotoxicity	5	PLCγ1, PLCγ2, SYK	6.45E-06
C	hsa04666:Fc gamma R-mediated phagocytosis	3	PLCγ1, PLCγ2, SYK	0.0049
	hsa04660:T cell receptor signaling pathway	3	ITK, PLCγ1, LCP2	0.0063
	hsa04670:Leukocyte transendothelial migration	3	ITK, PLCγ1, PLCγ2	0.0075
	hsa05310:Asthma	2	FCER1A, FCER1G	0.0338
D	hsa00230:Purine metabolism	6	ADCY2, NME1, PKLR	2.31E-08
	hsa00240:Pyrimidine metabolism	5	NME2, NME1	6.15E-05
	hsa03020:RNA polymerase	2	POLR3A, POLR3D	0.0272
	hsa04623:Cytosolic DNA-sensing pathway	2	POLR3A, POLR3D	0.0529
E	hsa04080:Neuroactive ligand-receptor interaction	7	C5AR1, PTGER3, FPR1	1.10E-05
	hsa04062:Chemokine signaling pathway	5	CCL25, ADCY2, CCL21, CXCR6, CXCL9	6.95E-04
F	hsa04060:Cytokine-cytokine receptor interaction	4	CCL25, CCL21, CXCR6, CXCL9	0.0210
	hsa04114:Oocyte meiosis	6		
	hsa04110:Cell cycle	6	CCNB1, CCNB2, MAD2L1, BUB1, ESPL1, AURKA CCNB1, CCNB2, MAD2L1, BUB1, PCNA, ESPL1	1.56E-05 2.91E-05
	hsa03050:Proteasome	4	PSMB10, PSMD14, PSMA6, PSMC4	3.81E-04
	hsa04914:Progesterone-mediated oocyte maturation	4	CCNB1, CCNB2, MAD2L1, BUB1	0.0022
